# Supplementary material for: Femtosecond control of phonon dynamics near a magnetic order critical point
Source: Nat Commun. 2021 May 17;12:2865. doi: 10.1038/s41467-021-23059-2 (PMC8129429; doi:10.1038/s41467-021-23059-2)
Supplement: Supplementary file 1 — Supplementary Information [file 41467_2021_23059_MOESM1_ESM.pdf]

Supplementary Information for

Femtosecond control of phonon dynamics near a magnetic order critical point

O. Yu. Gorobtsov[1†], L. Ponet[2,3†], S. K. K. Patel[4,5], N. Hua[4,5], A. G. Shabalin[4], S. Hrkac[4], J. Wingert[4], D. Cela[4], J. M. Glownia[6], D. Zhu[6], R. Medapalli[5,7], M. Chollet[6], E . E. Fullerton[5], S. Artyukhin[2*], O. G. Shpyrko[4,5], A. Singer[1*]

1 – Materials Science and Engineering Department, Cornell University, Ithaca, NY 14853, USA

2 – Central Research Labs, Italian Institute of Technology, Genova, Italy

3 - Scuola Normale Superiore, Pisa, Italy

4 – Department of Physics, University of California, San Diego, La Jolla, California, 92093, USA

5 - Center for Memory and Recording Research, University of California, San Diego, La Jolla, California, 92093, USA

6 - The Linac Coherent Light Source, SLAC National Accelerator Laboratory, Menlo Park, CA 94025 USA

7 - Department of Physics, School of Sciences, National Institute of Technology, Andhra Pradesh 534102, India

**Correspondence to: asinger@cornell.edu, sergey.artyukhin@iit.it*

† - These authors contributed equally to this work

Supplementary Note 1: Spin density wave behaviour in a thin chromium film compared to bulk

Here we describe the effect of dimensional confinement on SDW in chromium. Bulk Cr is an archetypal example of a material with an incommensurate spin density wave (SDW). The Fermi-surface nesting determines the SDW- wavevector Q: three SDWs with three orthogonal Q wavevectors exist in bulk. [1]. However, interfaces and dimensionality effects are well known to affect the SDW properties of Cr [2], and the nature of the effects has been extensively studied [3] [4] [5] [6] [7] [8]. The limited film thickness breaks the symmetry of the system and introduces boundary conditions. The SDW and the associated CDW and PLD are oriented normal to the film surface, and scattering experiments show no in-plane SDW [6] [9]. In Cr (001) films grown on MgO substrate (the substrate used in our experiment), the boundary conditions on the substrate/film border enforce a structure on SDW, CDW, and PLD, with the exact phase of the wave previously determined from X-ray measurements [7] [8] (see example in Supplementary Fig. 1). Since the PLD has a twice smaller period than SDW, the PLD wave vector $2Q$ is also quantized to a half-integer $N_P = (N + 1/2)$ number of PLD periods in the film. The exact number N_P depends on the temperature and the film thickness (see [7] for N_P measurements as a function of temperature for the film studied in this work).

Apart from the direction and quantization of the SDW, CDW, and PLD in a thin film, another important consideration is the effect of thickness on the Néel temperature. Upon reduction of one linear dimension to L the critical temperature $T_N(L)$ changes due to dimensional crossover according to a scaling law [10] [2]

$$\frac{T_N(L) - T_N(\infty)}{T_N(\infty)} \sim L^{-\lambda}, \qquad (1)$$

where λ is the shift exponent and λ = 1/ν, ν is the critical exponent for the correlation length. In bulk Cr, $T_N(\infty) = 311\ K$ [1]. The decrease in T_N at the lower film thicknesses has indeed been observed, and the Néel temperature lies approximately in the range $T_N = 290\ \pm 5\ K$ for Cr (001) films of similar thicknesses grown on the same substrate (MgO) as in our work [6] [9].

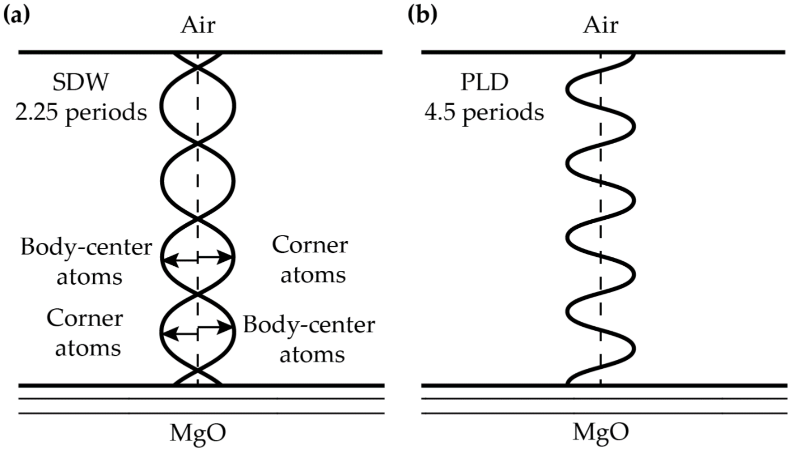

Supplementary Figure 1**. Interface pinning of SDW and corresponding PLD. a** An example of an allowed SDW in a Cr film on the MgO interface. **b** Corresponding PLD. The spatial period of PLD is twice shorter than SDW, so only half-integer period numbers are allowed.

Supplementary Note 2: X-ray diffraction from a thin film with a periodic lattice distortion

Here, we provide a detailed description of the X-ray diffraction from a thin crystalline film with a periodic lattice distortion (PLD) in one dimension, based mostly on the treatment in [7], [11]. We consider a thin homogeneous sample with [001] out-of-plane direction, as in our experiment, and correspondingly consider a PLD with a wave vector along $\boldsymbol{r} = [00r]$ and $\boldsymbol{q} = [00q]$. The scattered amplitude in the kinematic approximation can be written as [12]

$$F(q) = F_u(q) \sum_{n=0}^{N-1} e^{iqr_n}, \qquad (2)$$

where $F_u(q)$ is the structure factor of the unit cell, which includes atomic scattering, and the summation is performed over the crystal layers. In the presence of a PLD, the positions of the unit cells can be written as

$$r_n = r_n^0 + A_{PLD} cos(2Qr_n^0 - \phi_0), \qquad (3)$$

where $r_n^0 = n \cdot a$ are the undistorted positions, a is the lattice constant, A_{PLD} is the amplitude, $2Q$ is the wave vector of the PLD, and ϕ_0 defines the offset of the wave. Substituting Supplementary Eq. (2) in Supplementary Eq. (1), using the well-known expression

$$e^{iz\,cos(\phi)} = \sum_{k=-\infty}^{\infty} i^k J_k(z) e^{ik\phi}, \qquad (4)$$

with $J_k(z)$ being the Bessel function of k-th kind, and considering only terms of order qA_{PLD}, as qA_{PLD} is typically on the order of 10^{-2} [1], yields

$$F(q) = F_u(q)\sum_{n=0}^{N-1}\left(e^{iqr_n^0} + \frac{iqA_{PLD}}{2}\cdot\left[e^{i\phi_0}e^{i(q-2Q)r_n^0} + e^{-i\phi_0}e^{i(q+2Q)r_n^0}\right]\right). \quad (5)$$

All terms in Supplementary Eq. (5) can be readily evaluated [12], and using $f(q) = \frac{sin(Nqa/2)}{sin(qa/2)}$ the measured intensity can be written as

$$I(q) = |F_u(q)|^2\left[|f(q)|^2 - qA_{PLD}\,sin(\alpha)\,f(q)f(q+2Q) + qA_{PLD}sin(\alpha)f(q)f(q-2Q) + \frac{(qA_{PLD})^2}{4}|f(q+2Q)|^2 + \frac{(qA_{PLD})^2}{4}|f(q-2Q)|^2\right], \qquad (6)$$

where $\alpha = Qa[N-1] - \phi_0$ and interference terms between the satellites $f(q-2Q)f(q+2Q)$ have been neglected, since $f(q)$ decays rapidly from its central peak. Bragg peaks are at $q = G_{00l} = 2\pi l/a$ with integer l and the PLD peaks are located around the Bragg peaks at $q = G_{00l} \pm 2Q$.

In a bulk crystal, $f(q)$ can be approximated as a Dirac δ function. In this case, the interference terms $f(q)f(q+2Q)$ and $f(q)f(q-2Q)$ can be neglected, and the PLD results in a well-known configuration [1], with clear satellite peaks at $(G_{00l} - 2Q)$ and $(G_{00l} + 2Q)$ (see Supplementary Fig. 2, a). However, in a thin film with limited N (~90 for 30 nm Cr film), the satellite positions are still within the main peak, and interference terms dominate the intensity at the satellite positions, while the term $|f(q-2Q)|^2$ can be neglected in comparison. An example of the resulting $I(q)$ is illustrated in Supplementary Fig. 2, b, with the intensity increased at $G_{00l} - 2Q$ and decreased at $G_{00l} + 2Q$. It has been previously observed experimentally, for example, in [8]. The change in the satellite intensity is then linearly proportional to A_{PLD}, as seen from Supplementary Eq.(6) and discussed in the main text.

The pinning of the density wave on both surfaces of the film, discussed in the previous section, means that parameter α can be expressed as $\alpha = N_P\pi - \phi_0$, where N_P is the number of PLD periods in the film. The quantized nature of the PLD wave vector Q as a function of film thickness means that the position of the satellite will be correlated with the positions of Laue diffraction fringes (see [8]). In our experiment (T=118 K, N~90) the value of $N_P = 8.5$ and the satellite intensity appears at the 8th fringe.

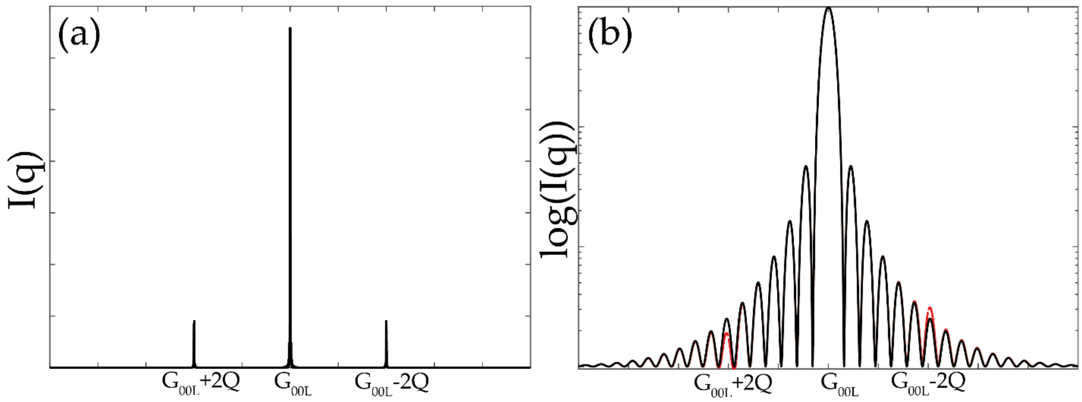

Supplementary Figure 2. **The qualitative difference in X-ray diffraction from a bulk crystal and a thin film and with a PLD. a** In bulk, sharp satellite peaks are observed around the Bragg peak at positions corresponding to the PLD wave vector. **b** In a thin film, interference between PLD scattering and the Laue fringes leads to destructive interference on one side and constructive on the other side. The satellite position coincides with the position of a fringe because of the PLD quantization defined by the film thickness.

Supplementary Note 3: The geometric configuration of the X-ray diffraction peak

We performed the measurements near the (002) out-of-plane peak in Bragg geometry (Supplementary Fig. 3, a) in the same manner as described previously in [11] (see Supplementary Materials in [11]). The signal recorded on the area detector (see Fig. S2, c) represents a cross section of the Bragg peak with the Ewald sphere (see Supplementary Fig. 3, b) through the Bragg rod oriented normal to the film surface. The Ewald sphere is angled to the Bragg rod at the Bragg angle θ_B =30 deg. By adjusting the rocking angle, we positioned the maximum of the PLD fringe $G_{002} - 2Q$, which shows constructive interference discussed in the previous section, on the detector. In the diffraction pattern (Supplementary Fig. 3, c), the PLD fringe and the neighbouring fringes still lying on the Ewald sphere are visible. The Ewald sphere still intersects an edge of the main Bragg peak, which contains enough intensity to also appear on the detector (Supplementary Fig. 3, c, far left) and serve as a reference point.

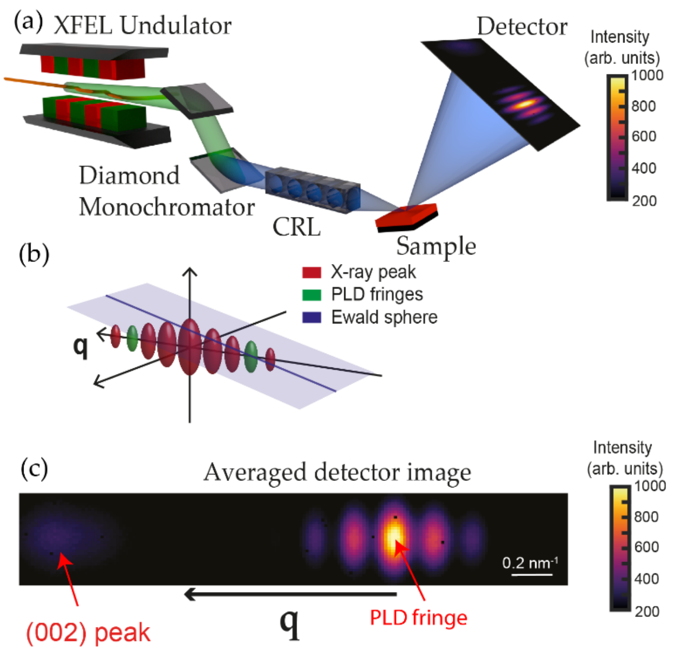

Supplementary Figure 3. **X-ray diffraction geometry. a** Scheme of the beamline setup. X-ray beam from the undulator is monochromatized, focused to 0.2x0.2 mm^2 size by a compound refractive lens (CRL), and then scatters from the sample in reflection (Bragg) geometry. **b** X-ray scattering from periodic atomic displacement is detected on Laue fringes of the main peak from the crystalline film. **c** Average static diffraction pattern observed in the experiment, with a central peak, PLD fringe and neighbouring fringes visible.

Supplementary Note 4: Data collection and analysis

Data collection is performed using the so-called LCLS timetool [13]: the arrival times of the pulses are randomly distributed (with a uniform probability distribution) within the chosen delay range and measured with a sub-10 fs precision, and the diffraction patterns are averaged within 30 fs intervals. Unlike data collection at fixed time delays, the random distribution approach minimizes the errors due to the temporal jitter and the probing frequency. On average ~200 patterns per 30 fs interval have been collected for 1D delay scans and ~30 patterns per 30x30 fs interval for 2D delay scans. Several 2D delay scans were combined to obtain the total pattern in Fig. 2. The high number of patterns is necessary to decrease the error coming from the stochastic nature of the SASE emission (the intensity varies by almost 100 % because of the monochromator used). In order to estimate the measurement precision, we calculate the standard deviation before the arrival of the laser pulse. The result of the measurement is a time-dependent intensity distribution with Laue fringes as in Supplementary Fig. 3, b.

The change in intensity of a Laue fringe due to the interference with the PLD signal is directly proportional to the PLD amplitude A_{PLD} (see Supplementary Eq. (6)). After the

photoexcitation quenches the spin order and the PLD amplitude starts to change (acoustic phonon), the intensity in the corresponding fringe will change as well. The excited acoustic phonon with the same spatial period as the PLD leads to the observed oscillation (Supplementary Fig. 3, a) at the expected fringe (8th). A total intensity $I_{ROI}(A_{PLD})$ in the region of interest (ROI) depicted by a red rectangle is chosen to track the changes in the fringe associated with A_{PLD} change. After subtracting the scattering background, we need to subtract the intensity without the PLD $I_{ROI}(A_{PLD}=0)$. As observed before in [11] (see for ex. Fig. 4, c in [11]), at the laser fluences above 4 mJ/cm^2 the minimum observed value of $A_{PLD}(t)$ reaches saturation, and the $I_{ROI}(A_{PLD}=0)$ is observed at 0.11 ps after the photoexcitation (see [11]) giving a convenient way to find $I_{ROI}(A_{PLD}=0)$ from measurements at high-laser fluences. The value of $I_{ROI}(A_{PLD}=0)$ obtained in such a way is then independent of the laser fluence and can be used for all measurements at the same starting temperature (T=115 K). After subtracting the $I_{ROI}(A_{PLD}=0)$, the remaining I_{ROI} is then normalized to 1 before the photoexcitation, and the resulting $\left(I_{ROI}\left(A_{PLD}(t)\right) - I_{ROI}(A_{PLD}=0)\right)/I_{ROI}\left(A_{PLD}(t<0)\right) = A_{PLD}(t)/A_{PLD}(t<0)$ due to the already mentioned linear dependence.

Supplementary Note 5: Thermal effects and temperature estimation

Thermal expansion of the lattice moves the Bragg peak in the negative q direction after ~1 ps. However, the total movement of the fringe is ~1 pixel, which is <5% of the PLD fringe width for the 28 nm film. In order to estimate the final temperature of the system after thermalization within the film (but not with the substrate), we can rely on the final relative level of PLD after thermalization (at ~10 ps). The value of the PLD is approximately 0.7 of the value at 115 K; comparing this with for ex. [14], we can conclude that the final temperature of the lattice is approximately 250 K.

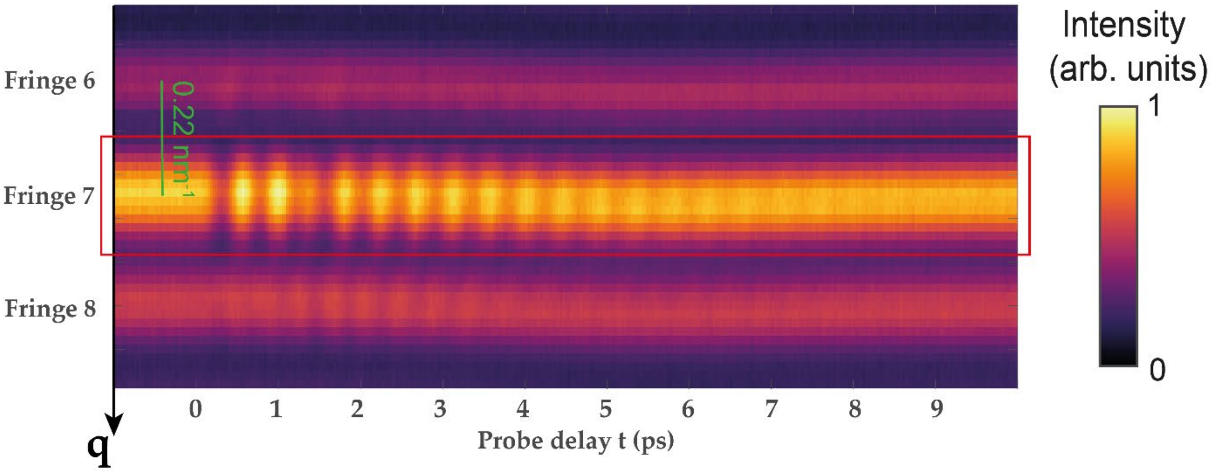

Supplementary Figure 4. **The intensity profile of the satellite peak as a function of the time delay.** Thermal expansion of the lattice leads to movement of the fringe to lower q.

Supplementary Note 6: Additional data

In addition to the data shown in the main text, we have also collected multiple high statistics datasets similar to the ones shown in Fig. 1 in the main text, at different delays between the pump pulses. We have collected several datasets showing clear enhancement of the acoustic phonon (Supplementary Fig. 5). Note that the enhancement is clearly observed independently of the number of the phonon temporal periods between the optical pulses. We have also achieved full suppression of the vibrational state at different delays (Supplementary Fig. 6). The suppression is again achieved at different

period numbers. Several datasets with intermediate behaviour have also been collected (Supplementary Fig. 7).

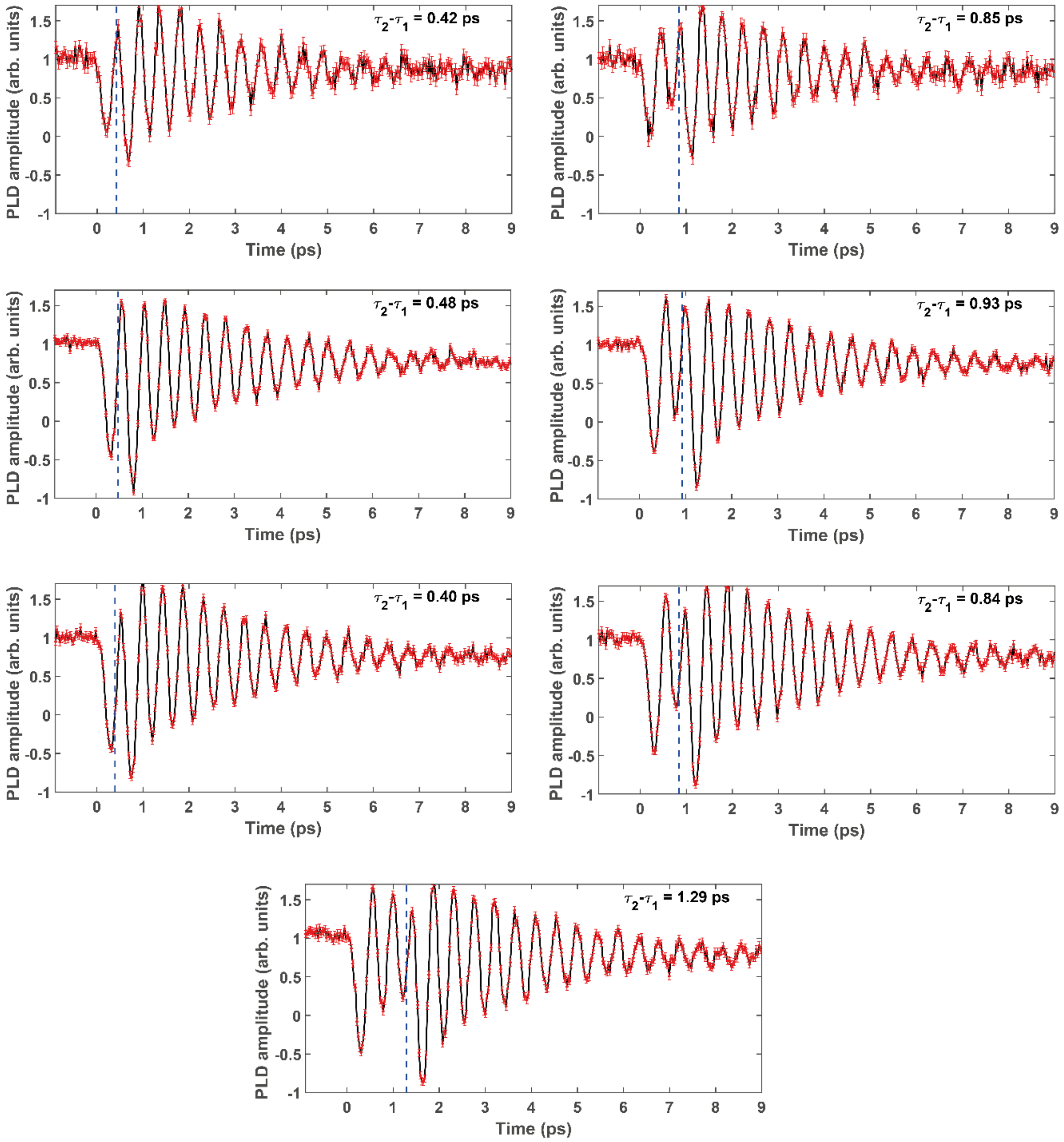

Supplementary Figure 5. **Enhancement of the acoustic phonon.** Additional data with different pump-pump delays showing enhancement of the acoustic phonon. Scans with $\tau = 0.42\ ps$ and $\tau = 0.85\ ps$ are outliers collected during calibration. Vertical bars show uncertainties, estimated as a standard deviation σ_{FEL} of the PLD amplitude at *t<0* and as $\sigma_{full} = \sqrt{\sigma_{FEL}^2 + \sigma_{ex}^2}$ at *t>0*, where σ_{ex} is a standard deviation of the minimum reached PLD amplitude after the first excitation between different scans with identical fluence P.

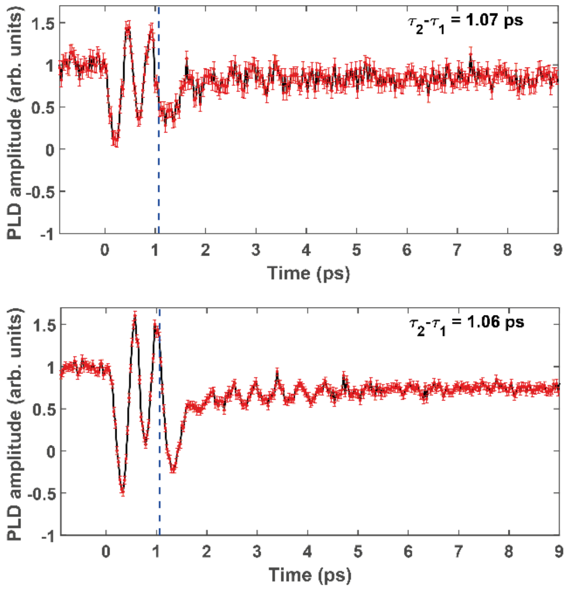

Supplementary Figure 6. **Suppression of the acoustic phonon.** Additional data with different pump-pump delays showing suppression of the acoustic phonon. Scan with $\tau = 1.07\ ps$ is an outlier collected during calibration. Vertical bars show uncertainties, estimated as a standard deviation σ_{FEL} of the PLD amplitude at *t<0* and as $\sigma_{full} = \sqrt{\sigma_{FEL}^2 + \sigma_{ex}^2}$ at *t>0*, where σ_{ex} is a standard deviation of the minimum reached PLD amplitude after the first excitation between different scans with identical fluence P.

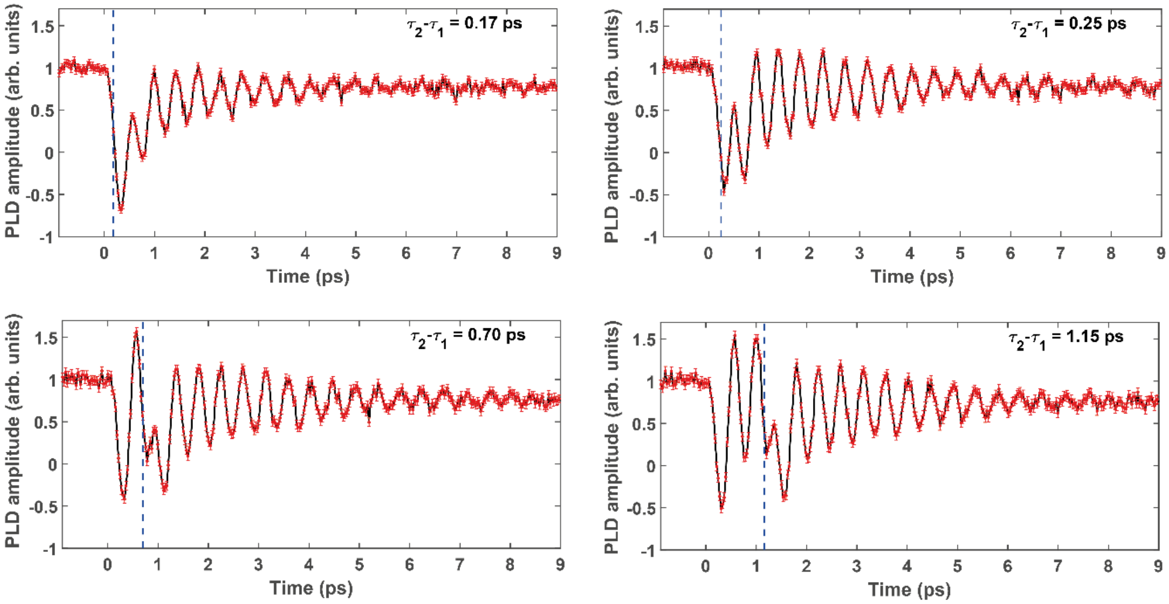

Supplementary Figure 7. **Change in the acoustic phonon.** Additional data with different pump-pump delays showing intermediate situations. Vertical bars show

uncertainties, estimated as a standard deviation σ_{FEL} of the PLD amplitude at *t<0* and as $\sigma_{full} = \sqrt{\sigma_{FEL}^2 + \sigma_{ex}^2}$ at *t>0*, where σ_{ex} is a standard deviation of the minimum reached PLD amplitude after the first excitation between different scans with identical fluence P.

Supplementary Note 7: On the absence of the topological defect removal timescale

We have verified that we do not observe a timescale associated with removal of topological defects. First, removal of topological defects would lead to a decrease of the disorder in the domain structure, causing an increase in the width of the scattering from the PLD and a corresponding decrease of the satellite X-ray peak intensity, which is not observed. Second, while the change in pulse intensity does cause a slight increase in the order recovery time [11], the increase is not as significant as in [15, 16, 17], and can instead be explained sufficiently by temperature difference [11] [18]. The absence of topological timescale in our data can be explained by a low film thickness that inhibits all but one SDW wavevector direction, causing the domains to not form in the "depth" of the film. Additionally, it suggests that the domain boundaries splitting the film surface are enforced extrinsically by defects in the structure.

Supplementary References

[1] E. Fawcett, "Spin-density-wave antiferromagnetism in chromium," *Reviews of Modern Physics,* vol. 60, p. 209–283, 1 1988.

[2] H. Zabel, "Magnetism of chromium at surfaces, at interfaces and in thin films," *Journal of Physics: Condensed Matter,* vol. 11, p. 9303–9346, 11 1999.

[3] A. M. N. Niklasson, B. Johansson and L. Nordström, "Spin Density Waves in Thin Chromium Films," *Physical Review Letters,* vol. 82, p. 4544–4547, 5 1999.

[4] E. E. Fullerton, S. D. Bader and J. L. Robertson, "Spin-Density-Wave Antiferromagnetism of Cr in Fe/Cr(001) Superlattices," *Physical Review Letters,* vol. 77, p. 1382–1385, 8 1996.

[5] E. E. Fullerton, J. L. Robertson, A. R. E. Prinsloo, H. L. Alberts and S. D. Bader, "Hysteretic Spin-Density-Wave Ordering in Confined Geometries," *Physical Review Letters,* vol. 91, 12 2003.

[6] R. K. Kummamuru and Y.-A. Soh, "Electrical effects of spin density wave quantization and magnetic domain walls in chromium," *Nature,* vol. 452, p. 859–863, 4 2008.

[7] A. Singer, M. J. Marsh, S. H. Dietze, V. Uhlíř, Y. Li, D. A. Walko, E. M. Dufresne, G. Srajer, M. P. Cosgriff, P. G. Evans, E. E. Fullerton and O. G. Shpyrko, "Condensation of collective charge ordering in chromium," *Physical Review B,* vol. 91, 3 2015.

[8] A. Singer, S. K. K. Patel, V. Uhlíř, R. Kukreja, A. Ulvestad, E. M. Dufresne, A. R. Sandy, E. E. Fullerton and O. G. Shpyrko, "Phase coexistence and pinning of charge density waves by interfaces in chromium," *Physical Review B,* vol. 94, 11 2016.

[9] Y.-A. Soh and R. K. Kummamuru, "Spintronics in antiferromagnets," *Philosophical Transactions of the Royal Society A: Mathematical, Physical and Engineering Sciences,* vol. 369, p. 3646–3657, 9 2011.

[10] M. N. Barber, Phase transitions and critical phenomena, vol. 8, C. Domb and J. L. Lebowitz, Eds., London, New: Academic Press, 1983, p. 148.

[11] A. Singer, S. Patel, R. Kukreja, V. Uhlíř, J. Wingert, S. Festersen, D. Zhu, J. Glownia, H. Lemke, S. Nelson, M. Kozina, K. Rossnagel, M. Bauer, B. Murphy, O. Magnussen, E. Fullerton and O. Shpyrko, "Photoinduced Enhancement of the Charge Density Wave Amplitude," *Physical Review Letters,* vol. 117, 7 2016.

[12] B. E. Warren, X-Ray Diffraction, DOVER PUBN INC, 1990.

[13] M. Harmand, R. Coffee, M. R. Bionta, M. Chollet, D. French, D. Zhu, D. M. Fritz, H. T. Lemke, N. Medvedev, B. Ziaja, S. Toleikis and M. Cammarata, "Achieving few-femtosecond time-sorting at hard X-ray free-electron lasers," *Nature Photonics,* vol. 7, p. 215–218, 2 2013.

[14] J. P. Hill, G. Helgesen and D. Gibbs, "X-ray-scattering study of charge- and spin-density waves in chromium," *Physical Review B,* vol. 51, pp. 10336-10344, 4 1995.

[15] A. Zong, A. Kogar, Y.-Q. Bie, T. Rohwer, C. Lee, E. Baldini, E. Ergeçen, M. B. Yilmaz, B. Freelon, E. J. Sie, H. Zhou, J. Straquadine, P. Walmsley, P. E. Dolgirev, A. V. Rozhkov, I. R. Fisher, P. Jarillo-Herrero, B. V. Fine and N. Gedik, "Evidence for topological defects in a photoinduced phase transition," *Nature Physics,* vol. 15, pp. 27-31, 10 2018.

[16] A. Zong, P. E. Dolgirev, A. Kogar, E. Ergeçen, M. B. Yilmaz, Y.-Q. Bie, T. Rohwer, I.-C. Tung, J. Straquadine, X. Wang, Y. Yang, X. Shen, R. Li, J. Yang, S. Park, M. C. Hoffmann, B. K. Ofori-Okai, M. E. Kozina, H. Wen, X. Wang, I. R. Fisher, P. Jarillo-Herrero and N. Gedik, "Dynamical Slowing-Down in an Ultrafast Photoinduced Phase Transition," *Physical Review Letters,* vol. 123, 8 2019.

[17] P. E. Dolgirev, M. H. Michael, A. Zong, N. Gedik and E. Demler, "Universal dynamics of order parameter fluctuations in pump-probe experiments," 6 10 2019.

[18] C. Nicholson, C. Monney, R. Carley, B. Frietsch, J. Bowlan, M. Weinelt and M. Wolf, "Ultrafast Spin Density Wave Transition in Chromium Governed by Thermalized Electron Gas," *Physical Review Letters,* vol. 117, 9 2016.

[19] E. Rotenberg, B. K. Freelon, H. Koh, A. Bostwick, K. Rossnagel, A. Schmid and S. D. Kevan, "Electron states and the spin density wave phase diagram in Cr(110) films," *New Journal of Physics,* vol. 7, p. 114–114, 4 2005.
